# Multimodal Nursing Care for Stroke Caregivers: Protocol for a Randomized Controlled Trial

**DOI:** 10.2196/88950

**Published:** 2026-11-13

**Authors:** Patricia Reddy, Ranjana Sharma

**Affiliations:** 1 Department of Medical Surgical Nursing, Shrimati Radhikabai Meghe Memorial College of Nursing and Shalinitai Meghe College of Nursing, Wardha, Datta Meghe Institute of Higher Education and Research, Sawangi (Meghe), Wardha, Maharashtra, 442107, India, 91 8380094958

**Keywords:** stroke, cerebrovascular accident, caregiver burden, multimodal intervention, randomized controlled trial, nursing care, progressive muscle relaxation, transitional care

## Abstract

**Background:**

Stroke is one of the most devastating neurological conditions worldwide, leaving survivors dependent on family members for long-term care. Family caregivers of stroke survivors in India frequently experience significant physical, emotional, social, and financial burdens, particularly during the hospital-to-home transition, with little structured support available. To address this gap, the Multimodal Approach to Nursing Care (MANC) module was developed as a structured nurse-led intervention combining caregiver education, medical device management training, psychosocial support, and progressive muscle relaxation techniques.

**Objective:**

This study aims to develop and validate the MANC module and evaluate its effectiveness in reducing caregiver burden and stress, and improving quality of life and caregiving competence among family caregivers of patients with cerebrovascular accident (CVA), compared with routine caregiving practices.

**Methods:**

This prospective randomized controlled trial will be conducted at the Acharya Vinoba Bhave Rural Hospital in Sawangi, Wardha, India. Ninety family caregivers of patients with CVA will be randomly allocated into experimental (n=45) and control (n=45) groups. Allocation concealment will be maintained through sequentially numbered, opaque, sealed envelopes. Given the single-researcher study design, complete blinding is not feasible. All outcome measures are standardized self-reported instruments, minimizing assessment bias. Outcomes include caregiver burden, stress, quality of life, caregiving competence, and patient health outcomes, assessed at baseline and postintervention. Data will be analyzed using appropriate parametric and nonparametric tests, with significance set at *P*<.05.

**Results:**

Ethical approval was obtained from the Institutional Ethics Committee, Datta Meghe Institute of Higher Education and Research (Deemed University) in December 2024 (IEC number DMIHER(DU)/IEC/2024/53), and the trial was registered with the Clinical Trials Registry–India (CTRI/2025/07/090412). Data collection commenced in February 2026, and recruitment is currently ongoing. Results are expected to be submitted for publication by the end of 2026.

**Conclusions:**

The MANC intervention is anticipated to reduce caregiver burden and stress while improving quality of life and caregiving competence among family caregivers of stroke survivors, potentially serving as a replicable model for poststroke transitional care across Indian tertiary care settings.

## Introduction

### Background

Stroke is a condition that occurs when blood flow to the brain is interrupted due to a blockage or bleeding, leading to brain cell death and serious neurological complications [1]. It is recognized as one of the leading causes of death and long-term disability worldwide, affecting individuals across all age groups and socioeconomic backgrounds [2]. The pathophysiology of stroke involves ischemic or hemorrhagic changes resulting in motor, cognitive, communicative, and behavioral deficits, necessitating comprehensive treatment strategies for a favorable recovery [3]. The close association between uncontrolled hypertension and stroke incidence further underlines the need for both preventive and rehabilitative approaches in stroke management [4]. In India, cerebrovascular accident (CVA) is a growing public health concern with a rising incidence across urban and rural populations, causing partial or complete disability and placing enormous demands on health care systems and families [2]. The Indian Stroke Association has emphasized, in its consensus statement on stroke rehabilitation, that structured postdischarge caregiver support is an essential component of comprehensive stroke care, underscoring the need for evidence-based nursing interventions targeting family caregivers [5].

When a family member suffers a stroke, a close relative is suddenly thrust into the demanding role of primary caregiver, often without prior preparation, adequate training, or emotional readiness. This transition places enormous physical and emotional demands on caregivers, fundamentally altering family dynamics. The quality of home-based care directly influences rehabilitation outcomes, complication rates, and hospital readmission rates. However, caregiving frequently brings significant physical, emotional, and financial stress that affects not only caregivers’ well-being but also patient recovery outcomes [6,7]. Sohkhlet et al [8] documented through an observational study that caregivers of stroke survivors undergoing rehabilitation experienced significant stress, with physical and emotional demands intensifying as rehabilitation progressed. Panicker et al [9] assessed caregiver burden among caregivers of stroke survivors at a rural-based medical teaching hospital in Western India and found that the majority experienced moderate-to-severe burden with limited institutional support—a finding that closely mirrors the context of the present study. In low-resource settings like India, where formal caregiver support programs remain largely inaccessible, these challenges are further compounded, making structured caregiver interventions a pressing necessity.

The burden experienced by family caregivers of stroke survivors has been documented extensively. Kochuvilayil and Varma [10] conducted a mixed methods study in Kerala among 221 palliative care caregivers using the EuroQol (EQ-5D-5L) and found that caregivers experiencing burden had the lowest mean quality-of-life score of 0.877 (SD 0.066), concluding that suitable interventions must be developed to target caregiver problems. Gaur et al [11] assessed the repercussions of caregiving among caregivers of stroke survivors and found that the majority experienced moderate-to-severe burden, with female caregivers and those in prolonged caregiving reporting the highest stress levels, emphasizing the urgent need for structured caregiver support programs. Kumar [12] conducted a narrative review on long-term caregiving and found that prolonged caregiving is associated with stress, burnout, and reduced quality of life. The study proposed the CARE-WELL model as a multidimensional framework for caregiver support, concluding that integrated caregiver-centered interventions are essential. Thayyil et al [13] evaluated a psychoeducation program at the National Institute of Mental Health and Neuro Sciences (NIMHANS) in Bengaluru among 35 caregivers of stroke survivors and demonstrated significant improvements in caregiver knowledge and preparedness, concluding that structured psychoeducation programs are effective in enhancing caregiver understanding.

Pogrebnoy et al [14] conducted a scoping review of 18 studies examining technology-based interventions in inpatient stroke rehabilitation and found that such approaches were generally feasible and acceptable, though further long-term research was recommended. Bakas et al [15], in their systematic review of 18 caregiver and dyad interventions for stroke survivors, found positive outcomes in reducing burden and improving quality of life across psychoeducation, skill-building, and counseling interventions, though methodological variability limited definitive conclusions, highlighting the need for rigorously designed randomized controlled trials (RCTs). A report on transitional care emphasized that inadequate discharge planning significantly increases caregiver burden and hospital readmissions, and that multidimensional nurse-led transitional care models improve patient outcomes and caregiver preparedness [16]. Shaji et al [17] conducted a randomized trial offering home-based support for dementia caregivers in Goa, reporting improved coping and reduced stress, while Mehta et al [18] demonstrated that the iSupport digital caregiver training platform improved caregiver knowledge and preparedness in the Indian context.

Evidence also strongly supports the inclusion of progressive muscle relaxation (PMR) as a caregiver support strategy. Çapacı et al [19] conducted an RCT evaluating PMR among caregivers of older patients and reported significant improvements in caregiver satisfaction, burden, and depression. Wang et al [20] evaluated a combined education and muscle relaxation program for caregivers of acute stroke survivors and reported significant reductions in anxiety, depression, and care burden, further validating the multimodal approach. George et al [21] similarly reported that a nurse-led PMR intervention significantly reduced stress, anxiety, and depression among caregivers in an RCT, demonstrating the broader applicability of PMR as a nurse-administered stress reduction strategy.

Collectively, the available evidence suggests that caregivers of stroke survivors experience significant physical, psychological, social, and economic burden, particularly during the hospital-to-home transition. Although psychoeducation programs, caregiver training modules, transitional care interventions, and PMR-based approaches have shown promise individually, many existing interventions remain fragmented, addressing isolated components without meeting the full spectrum of caregiver needs. There is a particular gap in interventions that simultaneously integrate caregiver education, medical device management training, psychosocial support, and stress reduction strategies within a single program. Therefore, this RCT proposes the Multimodal Approach to Nursing Care (MANC) module—a nurse-led intervention designed to equip family caregivers of patients with CVA with the knowledge, skills, and psychological resilience required for effective home-based caregiving.

### Study Objectives

The objectives of this study are as follows:

- To design and establish the validity of a MANC module intended for family caregivers of patients diagnosed with CVA;
- To evaluate current caregiving practices adopted by family caregivers of patients with CVA discharged from a selected hospital setting, and to examine the effectiveness of the MANC module compared with routine caregiving practices; and
- To examine the relationship between changes in caregiver-related outcomes and the corresponding health outcomes of patients with CVA among participants receiving the MANC intervention.

### Hypothesis

The study was guided by the following null and alternative hypotheses:

- Null hypothesis (H0): The MANC intervention will have no effect on caregiver burden, stress, quality of life, or caregiving competence among family caregivers of patients with CVA compared with routine caregiving practices.
- Alternative hypothesis (H1): The MANC intervention will have positive effects on caregiver burden, stress, quality of life, and caregiving competence among family caregivers of patients with CVA compared with routine caregiving practices.

## Methods

### Study Design

The present study is designed as a prospective RCT to evaluate the effectiveness of the MANC module among family caregivers of patients diagnosed with CVA. This protocol has been prepared and reported in accordance with the SPIRIT (Standard Protocol Items: Recommendations for Interventional Trials) 2013 guidelines [22]. The SPIRIT checklist has been completed and is provided as a supplementary file alongside this manuscript (Checklist 1). The intervention components of the MANC module are reported following the TIDieR (Template for Intervention Description and Replication) checklist to ensure full transparency regarding educational materials, device training procedures, psychosocial support sessions, PMR procedures, intervention dose, provider qualifications, and fidelity monitoring [22,23].

### Study Setting

The study will be conducted at Acharya Vinoba Bhave Rural Hospital (AVBRH), Sawangi, Wardha, in Central India, a tertiary care teaching hospital affiliated with Datta Meghe Institute of Higher Education and Research (Deemed University; DMIHER [DU]). Family caregivers of patients with CVA discharged from the neurology, medicine, and rehabilitation-related units of this hospital will be recruited for participation in the study. The hospital serves a large catchment population from rural and semiurban areas of the Vidarbha region of Maharashtra, making it an appropriate setting for studying caregiver burden in a resource-limited context.

### Ethical Considerations

The study protocol has been registered with the Clinical Trials Registry–India (CTRI), vide registration number CTRI/2025/07/090412, and ethical approval has been obtained from the Institutional Ethics Committee (IEC) of DMIHER(DU), Sawangi, Wardha, vide IEC approval number DMIHER(DU)/IEC/2024/53, granted on December 20, 2024. Prior to enrollment, written informed consent will be obtained from all participants.

### Eligibility Criteria

Careful attention was given to defining the eligibility criteria for this study to ensure that recruited participants are truly representative of the target population—family caregivers who are actively and primarily responsible for the home-based care of patients with CVA following hospital discharge (Textbox 1).

Textbox 1. **Eligibility criteria.**

**Inclusion criteria**

- Family caregivers who are the primary caregivers of patients diagnosed with cerebrovascular accident
- Caregivers aged 18 years and older
- Caregivers who are directly involved in providing home-based care following hospital discharge
- Individuals willing to participate in the Multimodal Approach to Nursing Care (MANC) intervention, including educational sessions, psychological support, and progressive muscle relaxation (PMR) techniques
- Caregivers who provide written informed consent for participation in the study

**Exclusion criteria**

- Professional or paid caregivers involved in patient care
- Caregivers of patients with severe comorbid illnesses requiring highly specialized care beyond the scope of this study
- Caregivers unwilling to participate in any component of the MANC intervention, including PMR
- Caregivers who are unable to understand the language used for intervention delivery and study communication
- Caregivers residing in geographically distant locations that may interfere with regular follow-up and intervention participation
- Caregivers with terminal illness or severe health conditions limiting participation throughout the study duration
- Caregivers who are currently enrolled in other interventional research studies that may influence study outcomes

### Recruitment and Randomization

Participants will be enrolled consecutively as they are discharged from the neurology and medicine units of AVBRH until the required sample size is achieved. Following baseline assessment, participants will be individually randomized to either the experimental group receiving the MANC intervention or the control group receiving routine care, using a computer-generated random number sequence prepared by the principal researcher using Microsoft Excel prior to data collection commencement. Allocation concealment will be maintained through sequentially numbered, opaque, sealed envelopes prepared in advance and opened only at the point of enrollment after baseline assessments.

### Blinding

Given the behavioral and educational nature of the MANC intervention and the single-researcher study design, complete blinding of the principal researcher is not feasible in this trial. The principal researcher will be responsible for both intervention delivery to the experimental group and outcome data collection for both groups. However, all outcome measures used in this study are standardized, validated, self-reported instruments, which minimize the risk of assessment bias arising from researcher involvement. Participants in the control group will not be informed of the specific components of the MANC intervention received by the experimental group, thereby maintaining partial participant blinding. This limitation is acknowledged and will be reported transparently in the final trial report.

### Sample Size

The sample size for this study was estimated based on the findings of Farahani et al [24], who evaluated a supportive home-care nursing intervention for caregivers of stroke survivors and reported a postintervention caregiver burden mean score of 62.63 (SD 22.68) in the routine education group and 40.46 (SD 17.28) in the supportive home-care group. The pooled SD was calculated as approximately 20.09. Using the formula for comparison of 2 independent means with a 95% confidence level (*Z*α/2=1.96), 80% power (*Z*β=0.84), and an expected mean difference of 12 points, the minimum sample size was calculated as 44 participants per group. After rounding up to account for potential dropouts, the final sample size was set at 45 family caregivers per group, giving a total of 90 participants.

The detailed sample size calculation is provided in Multimedia Appendix 1. To account for potential dropouts, 5 additional participants per group will be recruited, bringing the total enrolled sample size to 50 per group and 100 participants overall. In the event of participant withdrawal or loss to follow-up, the last observation carried forward (LOCF) method will be used as the primary imputation strategy within the intention-to-treat (ITT) analysis framework. A per-protocol sensitivity analysis will also be conducted, including only those participants who completed all assessment time points and received the full intervention as planned. Reasons for dropout will be documented and reported transparently in the final trial report. Given that all follow-up assessments will be conducted through in-person home visits by the researcher, attrition is expected to remain minimal throughout the study period.

### Intervention—The MANC Module

The MANC module is a nurse-led intervention developed specifically for family caregivers of patients with CVA. It was designed based on a comprehensive review of existing literature on caregiver burden, transitional care, and multimodal support strategies and was subsequently validated through expert review prior to implementation. The module integrates 4 core components delivered over a structured schedule during the postdischarge period.

The first component focuses on caregiver education, wherein caregivers receive detailed, structured information about stroke, its causes, warning signs of complications, rehabilitation exercises, positioning, skin care, feeding techniques, and the importance of medication adherence. Sessions are conducted in the local language using illustrated booklets and demonstration techniques to ensure comprehension across varying literacy levels. The second component addresses medical device management training, equipping caregivers with hands-on skills for the safe and accurate use of devices commonly required in poststroke home care, including feeding tubes, urinary catheters, suction apparatus, and monitoring equipment. Practical demonstrations and return demonstrations are used to ensure caregiver competence before discharge. The third component provides psychosocial support, addressing the emotional and psychological needs of caregivers through structured counseling sessions, peer interaction opportunities, and guidance on identifying and accessing available community and institutional support systems. This component acknowledges that caregiver well-being is as important as technical caregiving competence. The fourth component incorporates PMR training, a well-established stress reduction technique that involves systematic tensing and relaxation of muscle groups to reduce physiological and psychological stress responses. Caregivers are trained in PMR during the intervention period and are encouraged to practice it independently at home on a daily basis.

The control group will receive standard routine care at AVBRH at discharge—written discharge summary, physician advice, dietitian guidance, and hospital counselor support. No structured caregiver education, device training, psychosocial support, or PMR will be provided after discharge until the next hospital visit. Since participants in both experimental and control groups are enrolled from the same hospital during the same recruitment period, steps have been taken to minimize contamination. MANC intervention sessions will be conducted in a designated area separate from general ward areas. Participants in both groups will be advised at enrollment not to discuss their caregiving experiences with other caregivers at the hospital or in the community during the study period.

### Prevention of Contamination

Since participants in both experimental and control groups are recruited from the same tertiary care hospital, a deliberate recruitment strategy has been adopted to prevent contamination between groups. Additionally, MANC intervention sessions will be conducted in a designated area separate from the general ward areas. Participants in both groups will be advised at enrollment not to discuss their caregiving training or support experiences with other caregivers at the hospital or in the community during the study period.

### Outcome Measures

The primary outcome is caregiver burden, assessed using the Zarit Burden Interview (ZBI)—22 items, scored from 0 to 88; higher scores indicate greater burden (0‐20, little or no burden; 21‐40, mild to moderate; 41‐60, moderate to severe; 61‐88, severe). Secondary outcomes include caregiver stress, assessed using the Kingston Caregiver Stress Scale (KCSS)—10 items, scored from 10 to 50; caregiver quality of life, assessed using the Adult Carers Quality of Life (AC-QoL) Questionnaire—40 items, 8 subscales; caregiving competence, assessed using the Module-Based Caregiver Competence Questionnaire—40 marks total (10 for device management and 30 for knowledge); and patient health outcomes, assessed using the Stroke-Specific Quality of Life (SS-QoL) Scale—49 items across 12 domains. All outcomes will be assessed at baseline (T0—at discharge) and postintervention (T1—4 wk after discharge) by the principal researcher.

### Primary Outcome

The primary outcome of this study is caregiver burden, assessed using the ZBI. The ZBI is a widely used and well-validated 22-item self-report instrument that measures the subjective burden experienced by family caregivers across physical, psychological, social, and financial domains. Each item is rated on a 5-point Likert scale ranging from 0 (never) to 4 (nearly always), with total scores ranging from 0 to 88. Higher scores indicate greater caregiver burden. Scores are interpreted as follows: 0 to 20 (little or no burden), 21 to 40 (mild-to-moderate burden), 41 to 60 (moderate-to-severe burden), and 61 to 88 (severe burden). The ZBI has demonstrated strong internal consistency (Cronbach α>0.85) and has been validated for use among Indian caregiver populations. A reduction in the ZBI score of 8 to 10 points is considered a clinically meaningful improvement based on existing literature.

### Secondary Outcomes

#### Caregiver Stress

Caregiver stress will be assessed using the KCSS, a validated 10-item instrument that measures stress experienced across 3 domains: caregiving issues (7 items), family issues (2 items), and financial issues (1 item). Each item is rated on a 5-point scale from 1 (no stress) to 5 (extreme stress), with total scores ranging from 10 to 50. Higher scores indicate greater caregiver stress. The KCSS has demonstrated good reliability and validity in community-dwelling caregiver populations and is sensitive to change following interventions.

#### Caregiver Quality of Life

Caregiver quality of life will be assessed using the AC-QoL Questionnaire, a 40-item validated instrument measuring caregiver quality of life across 8 subscales: support for caring, caring choice, caring stress, money matters, personal growth, sense of value, ability to care, and carer satisfaction. Each item is rated on a 4-point scale (“never, some of the time, a lot of the time, and always”). The AC-QoL provides both subscale scores and a total quality of life score, with higher scores on positively worded subscales indicating better quality of life.

#### Caregiving Competence

Caregiving competence will be assessed using the Module-Based Caregiver Competence Questionnaire, a researcher-developed and expert-validated instrument consisting of 2 components: (1) the Caregiver Device Management (CDM) checklist, a 10-item observational checklist assessing practical competence in managing medical devices commonly used in poststroke home care (Foley catheter and nasogastric tube), with each item scored as yes (1) or no (0), giving a maximum device management score of 10; and (2) a 30-item knowledge assessment covering stroke awareness (10 items), medical device management (10 items), and complication recognition and management (10 items), with each correct response scoring 1 mark, giving a maximum knowledge score of 30. The total caregiving competence score ranges from 0 to 40, categorized as follows: 0 to 15 (needs significant guidance), 16 to 25 (needs occasional support), 26 to 33 (manages most tasks independently), and 34 to 40 (highly competent).

The Module-Based Caregiver Competence Questionnaire was developed by the principal researcher through a systematic process. A comprehensive review of existing literature on stroke caregiving, device management, and caregiver competence was conducted to identify relevant content domains. Items were generated to cover 3 domains: stroke knowledge and awareness, medical device management competence, and complication recognition and management. The instrument was subjected to content validation by a panel of 7 experts comprising nursing faculty, neurologists, and rehabilitation specialists, with a Content Validity Index (CVI) of 0.80 or above set as the criterion for item retention. Following expert validation, the instrument was pretested with 10 family caregivers of stroke survivors who were not part of the main study to assess clarity, comprehensibility, and feasibility of administration. Minor modifications were made based on pretesting feedback. Internal consistency of the knowledge component will be assessed using Cronbach α prior to the commencement of the main study.

#### Patient Health Outcomes

Patient health outcomes will be assessed using the SS-QoL, a validated 49-item, disease-specific instrument measuring health-related quality of life among stroke survivors across 12 domains: energy, family roles, language, mobility, mood, personality, self-care, social roles, thinking, upper extremity function, vision, and work/productivity. Each item is rated on a 5-point scale, with domain and total SS-QoL scores calculated. Higher scores indicate better patient quality of life and functional recovery. The SS-QoL has demonstrated strong validity and reliability in stroke survivor populations and is sensitive to clinically meaningful changes over time.

### Assessment Schedule

All primary and secondary outcome measures will be administered at 2 time points: (1) baseline (T0)—at the time of patient discharge from the hospital, prior to the commencement of the MANC intervention, and (2) postintervention (T1)—approximately 4 weeks after discharge, at the time of the home visit by the researcher.

### Statistical Analysis Plan

Data will be analyzed using SPSS version 25.0 (IBM Corp). The primary outcome (ZBI) will be compared between groups at postintervention using analysis of covariance (ANCOVA) adjusted for baseline scores, following ITT principles. Secondary outcomes will each be analyzed separately using ANCOVA with baseline adjustment. Within-group changes will be assessed using paired 2-tailed *t* tests or the Wilcoxon signed-rank test. Between-group differences will be assessed using independent 2-tailed *t* tests or the Mann-Whitney *U* test. Bonferroni correction will be applied for multiple secondary outcomes (adjusted threshold, *P*<.01). Key covariates, including caregiver age, gender, education, relationship with the patient, and stroke severity, will be adjusted for where baseline imbalances exist. Missing data will be handled using LOCF. A per-protocol sensitivity analysis will also be conducted. Correlation between caregiver and patient outcomes will be assessed using Pearson or Spearman correlation. All analyses will be 2-tailed, with significance set at *P*<.05 for the primary outcome.

### Primary Analysis

The primary analysis will compare caregiver burden scores (ZBI) between the experimental and control groups at postintervention (T1), adjusted for baseline scores (T0), using an ANCOVA model. This baseline-adjusted between-group comparison will be the primary inferential test for the study. All randomized participants will be included in the primary analysis regardless of protocol adherence or dropout, following the ITT principle. This approach ensures that the randomization is preserved and that the risk of bias due to selective exclusion of participants is minimized.

### Secondary Analyses

Each secondary outcome measure—caregiver stress (KCSS), caregiver quality of life (AC-QoL), caregiving competence (Module-Based Questionnaire), and patient health outcomes (SS-QoL)—will be analyzed separately using ANCOVA, adjusting for the respective baseline scores. Within-group changes from baseline to postintervention will be assessed using a paired *t* test for normally distributed data or a Wilcoxon signed-rank test for nonnormally distributed data. Between-group differences at postintervention will be assessed using an independent *t* test or a Mann-Whitney *U* test, as appropriate, after checking for normality with the Shapiro-Wilk test.

Given that multiple secondary outcomes are being tested simultaneously, a Bonferroni correction will be applied to control for the risk of type I error due to multiple comparisons. The adjusted significance threshold for secondary outcomes will therefore be set at *P*<.01 (0.05 divided by the number of secondary outcomes tested).

### Adjustment for Covariates

Key caregiver and patient variables that may influence outcomes—including caregiver age, gender, relationship with the patient, duration of caregiving, educational level, and patient stroke severity—will be recorded at baseline and included as covariates in ANCOVA models if statistically significant baseline imbalances are identified between groups.

### Correlation Analysis

The relationship between changes in caregiver-related outcomes (burden, stress, quality of life, and competence) and patient health outcomes (SS-QoL scores) will be assessed using the Pearson correlation coefficient for normally distributed data or the Spearman rank correlation for nonnormally distributed data.

### Missing Data and Dropout Handling

In the event of missing data due to participant dropout or loss to follow-up, the LOCF method will be used as the primary imputation strategy for the ITT analysis. A per-protocol analysis will also be conducted as a sensitivity analysis, including only participants who completed all assessment time points and received the full intervention as planned. Reasons for dropout will be documented and reported transparently in the final manuscript. Given that the researcher will conduct in-person home visits for all follow-up assessments, attrition is expected to be minimal.

### Significance Level

All statistical tests will be performed at a 2-tailed significance level of *P*<.05 for the primary outcome, with the Bonferroni correction applied for secondary outcomes as described earlier (Table 1).

**Table 1.** Study timeline.

Activities	Month 1	Month 2	Month 3	Month 4	Month 5	Month 6
Protocol preparation and finalization	✔					
IEC^a^ approval	✔	✔				
Pilot testing and training		✔				
Patient recruitment		✔	✔	✔		
Intervention and data collection		✔	✔	✔	✔	
Follow-up			✔	✔	✔	
Data entry and data cleaning				✔	✔	
Statistical analysis					✔	
Report writing and thesis drafting					✔	✔
Final submission						✔

a IEC: Institutional Ethics Committee.

## Results

Data collection commenced in February 2026, and recruitment of participants is currently ongoing at Acharya Vinoba Bhave Rural Hospital, Sawangi, Wardha. Results of the trial are expected to be submitted for publication by the end of 2026. The final trial results will be reported in accordance with the CONSORT (Consolidated Standards of Reporting Trials) 2010 guidelines to ensure complete and transparent reporting of the RCT [25].

## Discussion

### Principal Findings

The present RCT has been designed with the primary anticipation that the MANC intervention will demonstrate a significant reduction in caregiver burden and stress among family caregivers of patients diagnosed with CVA discharged from a tertiary care hospital. Beyond burden reduction, the MANC intervention is hypothesized to bring about meaningful improvements in caregiver quality of life and caregiving competence, outcomes that are closely interrelated and collectively determine the overall effectiveness of home-based poststroke care. The rationale for these anticipated findings is grounded in the multidimensional nature of the MANC module itself, which simultaneously addresses educational deficits, practical skill gaps, psychosocial needs, and physiological stress responses—areas that existing fragmented interventions have addressed only in isolation. If the anticipated outcomes are achieved, this study will provide strong evidence that a structured, nurse-led multimodal intervention can meaningfully transform the caregiving experience for families of stroke survivors in the Indian health care context.

### Comparison to Prior Work

The anticipated findings of this study are consistent with and build upon a growing body of evidence supporting structured caregiver interventions in poststroke care. Kumar [12] conducted a narrative review on long-term caregiving and established that prolonged caregiving is associated with stress, burnout, chronic illness, and reduced quality of life. The study concluded that integrated caregiver-centered interventions are essential for improving caregiver well-being and long-term health outcomes. The present study directly responds to this call by proposing a validated intervention rather than a fragmented support approach. Thayyil et al [13] demonstrated at NIMHANS, Bengaluru, that a structured psychoeducation program significantly improved caregiver knowledge and preparedness among caregivers of stroke survivors, with partial retention at follow-up. Although their intervention focused primarily on the educational component, the MANC module extends this approach by integrating psychosocial support, medical device management training, and PMR techniques alongside education, thereby addressing a broader spectrum of caregiver needs within a single structured program.

Bakas et al [15], in their systematic review of 18 family caregiver and dyad interventions for stroke survivors published between 2016 and 2021, found that psychoeducation, skill-building, counseling, telehealth support, and stress management interventions yielded positive outcomes in reducing caregiver burden and improving quality of life. However, the review also noted significant methodological variability, small sample sizes, and inconsistent outcome measures across studies, limiting the strength of conclusions that could be drawn. The present RCT addresses these methodological gaps through its randomized controlled design, clearly defined primary and secondary outcomes, and allocation concealment. Gaur et al [11] similarly highlighted through their cross-sectional study that caregiving for stroke survivors substantially affects caregiver well-being, with female caregivers and those involved in prolonged caregiving reporting the highest levels of stress and burden. The MANC intervention specifically targets these high-risk caregiver groups by providing comprehensive support from the point of hospital discharge, when caregiving demands are at their most intense and caregivers are least prepared.

A systematic review and meta-analysis by Jammal et al [26] further confirmed that structured interventions targeting informal caregivers of stroke survivors significantly reduced burden, stress, and strain, reinforcing the need for evidence-based multimodal approaches such as the MANC intervention proposed in the present study.

Xu et al [27] demonstrated through a cross-sectional study that family resilience and social support significantly mediated caregiver burden and caregiving capacity among caregivers of stroke survivors, highlighting the importance of psychosocial components within caregiver support interventions—a principle that is central to the MANC module.

Pitthayapong et al [28] evaluated a community-based caregiver support program for poststroke survivors in Thailand and reported improvements in caregiver preparedness and reduced caregiver burden following structured support, suggesting that nurse-led community and home-based interventions hold significant promise for caregivers of stroke survivor populations in Asian health care contexts similar to that of India.

The importance of transitional care support has also been well established in the existing literature. A comprehensive report on transitional care models emphasized that inadequate discharge planning and poor continuity of care significantly increase caregiver burden, complications, and avoidable hospital readmissions [16]. The MANC module is inherently a transitional care intervention, bridging the gap between hospital discharge and stable home-based care by equipping caregivers with the knowledge, skills, and psychological resilience required at this critical juncture. In this respect, the present study aligns with and extends the transitional care framework by providing a replicable, nurse-led model that can be implemented in tertiary care settings across India.

### Strengths and Limitations

This study has several notable strengths that distinguish it from existing caregiver intervention research. First and foremost, the MANC module adopts a genuinely multimodal approach, integrating 4 distinct components within a single structured intervention: caregiver education, medical device management training, psychosocial support, and PMR. Unlike studies that target only one dimension of caregivers’ needs, the MANC module acknowledges that caregiver burden is a multidimensional phenomenon that requires a multidimensional response. Second, the module was developed through a systematic process and subjected to expert validation prior to implementation, ensuring that its content is both evidence-based and contextually appropriate for caregivers of patients with CVA in the Indian setting. Third, the RCT design used in this study represents the gold standard for evaluating intervention effectiveness, providing the highest level of evidence for causal inference. The use of computer-generated randomization, allocation concealment through sequentially numbered, opaque, sealed envelopes, and partial participant blinding given the single-researcher study design further strengthens the methodological rigor of the study and minimizes the risk of selection and detection bias.

Despite these strengths, certain limitations of this study must be acknowledged. The study is being conducted at a single tertiary care center—AVBRH, Sawangi, Wardha—which may limit the generalizability of the findings to other health care settings, regions, and populations. Although the hospital serves a large catchment population from the Vidarbha region of Maharashtra, the sociodemographic and cultural characteristics of this population may not be fully representative of caregivers of stroke survivor populations across India. Additionally, given the behavioral and educational nature of the MANC intervention, complete blinding of participants and intervention providers is not feasible, which introduces the possibility of performance bias. However, this limitation is inherent to all behavioral intervention trials and has been partly mitigated by using standardized, self-reported outcome measures. The findings of this study should, therefore, be interpreted within these contextual boundaries, and future multicentric trials across diverse settings are recommended to establish the broader applicability of the MANC intervention.

### Future Directions

The present study is anticipated to lay the groundwork for a broader program of research on structured nursing interventions for caregivers of stroke survivors in India. Should the MANC intervention demonstrate significant effectiveness in reducing caregiver burden and improving quality of life, future studies could explore its adaptation and implementation across multiple tertiary care centers in different regions of India to establish its generalizability. Long-term follow-up studies examining the sustained effects of the MANC intervention beyond the immediate postdischarge period would also be valuable, as caregiver burden is known to evolve over time as the demands and complexities of caregiving change. Furthermore, future research could explore the integration of technology-assisted delivery formats—such as mobile app–based modules or telehealth platforms—to extend the reach of the MANC intervention to caregivers in geographically remote or underserved areas where in-person structured support is difficult to provide.

### Dissemination Plan

The findings of this RCT will be disseminated through multiple channels to ensure that the evidence generated reaches both the scientific community and relevant stakeholders in nursing practice and health care policy. The results will be submitted for publication in peer-reviewed nursing and health care journals, with priority given to journals focused on stroke rehabilitation, caregiver support, and nursing interventions. In addition, findings will be presented at national and international nursing conferences to facilitate knowledge exchange and promote awareness of the MANC intervention among nursing professionals and researchers. The study findings will also be shared with the hospital administration and nursing faculty at DMIHER (DU), with the aim of informing institutional practices and potentially incorporating the MANC module into nursing education and training curricula for future nurses working in stroke and rehabilitation care settings.

### Conclusions

Stroke imposes a profound and lasting burden not only on survivors but equally on family members who step into the demanding roles of primary caregivers, often without preparation, training, or adequate support. Although the clinical management of stroke has advanced considerably over the years, the structured support of family caregivers during the critical hospital-to-home transition remains a significantly under-addressed area, particularly in resource-limited settings like India. The MANC module represents a deliberate and evidence-informed effort to fill this gap by providing caregivers with the education, practical skills, psychological support, and stress management techniques they need to provide effective and sustainable home-based care.

The RCT design of this study ensures that the evidence generated will be of the highest methodological quality, enabling meaningful conclusions to be drawn about the true effectiveness of the MANC intervention in comparison with routine caregiving practices. The anticipated outcomes—reduced caregiver burden and stress, improved quality of life, and enhanced caregiving competence—if achieved, will have significant implications for how nursing care is structured and delivered in poststroke transitional care settings across India. Beyond the immediate clinical benefits, a validated and effective MANC module could serve as a replicable model that can be adapted and scaled across tertiary care institutions, potentially transforming the standard of caregiver support in stroke rehabilitation.

Ultimately, this study reflects a broader recognition that caring for the caregiver is not a secondary concern but a fundamental component of comprehensive stroke care. When family caregivers are adequately supported, informed, and empowered, the quality of care they provide to stroke survivors improves, rehabilitation outcomes are enhanced, and the risk of caregiver burnout and patient complications is reduced. It is hoped that the findings of this trial will contribute meaningfully to the growing evidence base for structured nursing interventions in caregiver support and inspire further research, policy attention, and institutional commitment to the well-being of family caregivers of stroke survivors in India and beyond.

## Supplementary material

10.2196/88950 Multimedia Appendix 1 Sample size calculation for the randomized controlled trial.

10.2196/88950 Checklist 1 SPIRIT checklist.

## Abbreviations

AC-QoL: Adult Carers Quality of Life
ANCOVA: analysis of covariance
AVBRH: Acharya Vinoba Bhave Rural Hospital
CDM: Caregiver Device Management
CONSORT: Consolidated Standards of Reporting Trials
CTRI: Clinical Trials Registry–India
CVA: cerebrovascular accident
CVI: Content Validity Index
DMIHER (DU): Datta Meghe Institute of Higher Education and Research (Deemed University)
IEC: Institutional Ethics Committee
ITT: intention-to-treat
KCSS: Kingston Caregiver Stress Scale
LOCF: last observation carried forward
MANC: Multimodal Approach to Nursing Care
NIMHANS: National Institute of Mental Health and Neuro Sciences
PMR: progressive muscle relaxation
RCT: randomized controlled trial
SPIRIT: Standard Protocol Items: Recommendations for Interventional Trials
SS-QoL: Stroke-Specific Quality of Life
TIDieR: Template for Intervention Description and Replication
ZBI: Zarit Burden Interview
