# Supplementary material for: Multimodal Nursing Care for Stroke Caregivers: Protocol for a Randomized Controlled Trial
Source: JMIR Res Protoc. 2026 Nov 13;15:e88950. doi: 10.2196/88950 (PMC13762683; doi:10.2196/88950)
Supplement: Supplementary file 1 [file resprot-v15-e88950-s001.docx]

**Sample Size Calculation**

The sample size for this study was estimated based on the findings of Farahani MA et al., who reported a post-intervention caregiver burden score of 62.63 ± 22.68 in the routine education group and 40.46 ± 17.28 in the supportive home-care group.

Pooled standard deviation = approximately 20.09

Formula applied:
n = 2 (Zα/2 + Zβ)² σ² / d²

Where:

- Zα/2 = 1.96 (95% confidence level)
- Zβ = 0.84 (80% power)
- σ = 20.09 (pooled standard deviation)
- d = 12 (expected mean difference)

n = 2 × (2.80)² × (20.09)² / (12)²
n = approximately 39

To ensure adequate statistical power and account for potential dropouts and variability in recruitment, the sample size was conservatively rounded up to 45 participants per group, giving a total of 90 participants. An additional 5 participants per group will be enrolled as a dropout buffer, bringing total planned enrolment to 100 participants.
