# Supplementary material for: Multimodal Nursing Care for Stroke Caregivers: Protocol for a Randomized Controlled Trial
Source: JMIR Res Protoc. 2026 Nov 13;15:e88950. doi: 10.2196/88950 (PMC13762683; doi:10.2196/88950)
Supplement: Supplementary file 2 [file resprot-v15-e88950-s002.docx]

**SPIRIT 2013 Checklist**

**Manuscript: Multimodal Nursing Care for Stroke Caregivers: A Randomized Controlled Trial Protocol**

MS#88950 — JMIR Research Protocols

Authors: Ms. Patricia Santosh Reddy, Dr. Ranjana Sharma

| **Item No.** | **SPIRIT 2013 Item** | **Location in Manuscript** |
| --- | --- | --- |
| **ADMINISTRATIVE INFORMATION** | | |
| 1 | Title — identifying the study as a randomized trial | Title Page |
| 2a | Trial registration number and registry name | Title Page; Results section — CTRI/2025/07/090412 |
| 2b | WHO trial registration data set | Title Page |
| 3 | Protocol version and date | Version 1, June 2026 |
| 4a | Funding sources | Funding Statement (end of manuscript) |
| 4b | Role of funder | Funding Statement — No external funding |
| 5a | Author names and affiliations | Title Page |
| 5b | Corresponding author contact | Title Page — patriciareddy4@gmail.com |
| 5c | Role of sponsor | N/A — no external sponsor |
| **INTRODUCTION** | | |
| 6a | Background and rationale | Introduction section |
| 6b | Explanation of trial design choice | Methods — Study Design section |
| 7 | Specific objectives and hypotheses | Introduction — Study Objectives and Hypothesis |
| **METHODS — PARTICIPANTS** | | |
| 8 | Trial design description | Methods — Study Design section |
| 9 | Study setting and location | Methods — Study Setting section (AVBRH, Sawangi, Wardha) |
| 10 | Eligibility criteria | Methods — Eligibility Criteria (double column table) |
| **METHODS — INTERVENTIONS** | | |
| 11a | Interventions for each group | Methods — Intervention (MANC Module) section; Control Group section |
| 11b | Criteria for modifying interventions | N/A — structured intervention with fixed protocol |
| 11c | Adherence strategies | Methods — personal home visits by researcher for follow-up |
| 11d | Concomitant care permitted | Methods — control group receives routine hospital discharge care |
| **METHODS — OUTCOMES** | | |
| 12 | Primary and secondary outcome measures | Methods — Outcome Measures section |
| 13 | Participant timeline | Methods — Study Timeline (Gantt chart) |
| 14 | Sample size calculation | Methods — Sample Size section |
| **METHODS — ASSIGNMENT** | | |
| 15 | Sequence generation | Methods — Recruitment and Randomization — computer-generated random number sequence |
| 16a | Allocation concealment | Methods — sequentially numbered opaque sealed envelopes |
| 16b | Allocation mechanism | Methods — prepared by independent statistician |
| 17 | Blinding | Methods — Blinding section |
| **METHODS — DATA COLLECTION** | | |
| 18a | Data collection tools and procedures | Methods — Outcome Measures section (ZBI, KCSS, AC-QoL, Module-Based Questionnaire, SS-QOL) |
| 18b | Plans to promote retention | Methods — personal home visits by researcher minimize attrition |
| 19 | Data management | N/A — to be detailed in full trial report |
| 20 | Statistical methods | Methods — Statistical Analysis Plan section |
| **METHODS — MONITORING** | | |
| 21a | Composition of coordinating centre | N/A — single centre study; researcher personally oversees data collection |
| 21b | Composition of data monitoring committee | N/A — doctoral research study; overseen by PhD supervisor Dr. Ranjana Sharma |
| 21c | Interim analyses and stopping guidelines | N/A — not applicable for this study given sample size and duration |
| 22 | Adverse events reporting | N/A — behavioral/educational intervention; no adverse events anticipated |
| 23 | Auditing | N/A — study audited through institutional PhD monitoring process |
| **ETHICS AND DISSEMINATION** | | |
| 24 | Research ethics approval | Results section — DMIHER(DU)/IEC/2024/53, December 2024 |
| 25 | Protocol amendments | N/A — Version 1; no amendments to date |
| 26 | Consent or assent | Methods — written informed consent obtained from all participants |
| 27 | Confidentiality | N/A — to be detailed in full trial report |
| 28 | Declaration of interests | Conflicts of Interest section (end of manuscript) |
| 29 | Access to data | N/A — data access restricted to research team |
| 30 | Ancillary and post-trial care | N/A — not applicable |
| 31a | Plans for dissemination | Discussion — Dissemination Plan section |
| 31b | Authorship | Title Page; Authors' Contributions section |
| 31c | Reproducible research | N/A — data sharing plan to be detailed in full trial report |

*Note: N/A = Not Applicable. All section references correspond to the submitted manuscript version.*
